# Supplementary material for: Functionally Validating Evolutionary Conserved Risk Genes for Parkinson’s Disease in Drosophila melanogaster
Source: Insects. 2023 Feb 9;14(2):168. doi: 10.3390/insects14020168 (PMC9958964; doi:10.3390/insects14020168)
Supplement: Supplementary file 1 [file insects-14-00168-s001.zip › Supplementary Figure S1, Tables S2-S9.pdf]

This file encompasses the below supplementary information associated with the manuscript.

Supplementary Figures

Figure S1	The rapid iterative negative geotaxis (RING) apparatus.

Supplementary Tables

Table S1	Overview PD associated genes and their *Drosophila* orthologs. [Excel file]
Table S2	PD risk genes and their evolutionary conserved genes in *D. melanogaster.*
Table S3	RNA sequencing metrics.
Table S4	Fold change of gene expression knockdown.
Table S5	Statistics on age-effect.
Table S6	Comparison of RNAi knockdown and common control at day 5.
Table S7	Comparison of RNAi knockdown and common control at day 10.
Table S8	Comparison of RNAi knockdown and common control at day 15.
Table S9	Survival data.
Table S10	Phenotypic data from the RING assay. [Excel file]

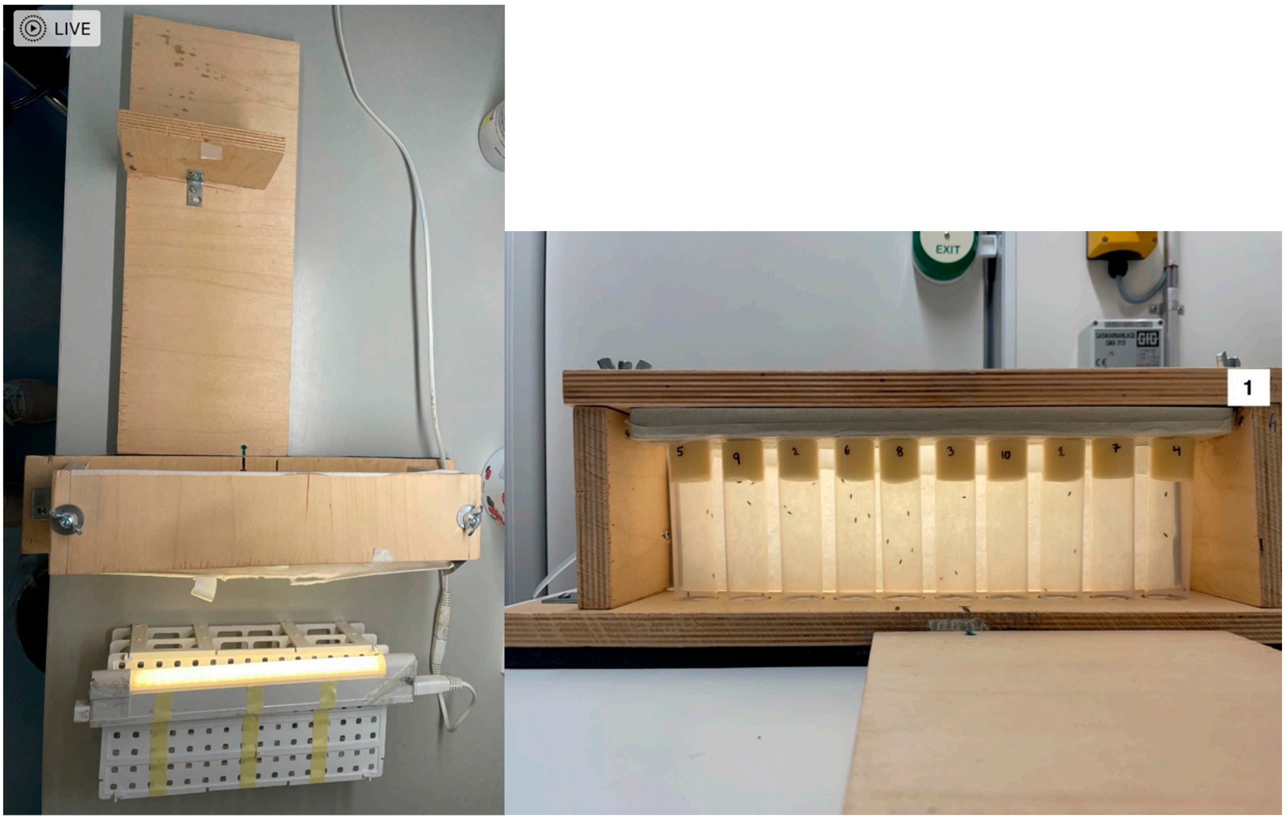

Supplementary Figure S1 ǀ Overview of the rapid iterative negative geotaxis (RING) system. The RING system consists of a wooden box with an open face and a removable lid held in place with hinges on each side, fastened using a bolt and a nut. The floor of the apparatus supports ten 46.63 mL vials (25 mm x 95 mm) (VWR™, PA) with a flat bottom. The lid is lined with foam to ensure that each tube is fastened in the apparatus. Adjacent to the face of the apparatus is a wooden board with a vertical board attached, allowing placement of a camera apparatus 30 cm in front of the RING apparatus. The camera apparatus was fastened to the wooded board using masking tape. To provide uniform lightning of the RING apparatus, a light source was placed behind a white opaque filter attached to the backside of the apparatus.

Supplementary Table S2 | Overview of the 11 selected PD risk genes with DIOPT score ≥12 and where a homozygous viable UAS line was available from VDRC. Complete overview of all PD associated genes can be seen in Supplementary Table S2.

PD risk gene	Reference	Fly gene symbol	VDRC strain	DIOPT score	UAS genotype
CLCN3	Nalls et al. 2019	*ClC-c*	6465	12	w^{1118}; P{GD1663}v6465
SLC44A1	Nalls et al. 2019<br>Chang et al. 2017	*Ctl1*	12242	14	w^{1118}; P{GD3579}v12242
HIP1R	Iwaki et al. 2019	*Hip1*	16137	12	w^{1118}; P{GD7152}v16137
ATP6V0A1	Chang et al. 2017	*Vha100-1*	22924	13	w^{1118}; P{GD12710}v22924
SH3GL2	Nalls et al. 2019<br>Chang et al. 2017	*EndoA*	24617	12	w^{1118}; P{GD7681}v24617
MCCC1	Nalls et al. 2019<br>Foo et al. 2020<br>Chang et al. 2017	*Mccc1*	25406	15	w^{1118}; P{GD9729}v25406
RPS6KL1	Nalls et al. 2019	*CG7156*	26035	12	w^{1118}; P{GD10657}v26035
MICU3	Chang et al. 2017	*MICU3*	41259	14	w^{1118}; P{GD5587}v41259
FAM49B	Nalls et al. 2019	*CG32066*	44825	12	w^{1118}; P{GD8705}v44825
CTSB	Nalls et al. 2019	*CtsB1*	45345	14	w^{1118}; P{GD6407}v45345
SLC41A1	Nalls et al. 2019<br>Chang et al. 2017<br>Iwaki et al. 20019	*CG33181*	47868	13	w^{1118}; P{GD16951}v47868
-	-	Control	60000	NA	w^{1118}

Supplementary Table S3 | QC metrics from BGI. After sequencing, the raw reads were filtered. Data filtering includes removing adaptor sequences, contamination, and low-quality reads from raw reads. Clean reads: Total clean reads number. Clean bases: Total clean bases number. Q20: The number of nucleotide with quality higher than 20/nucleotide(clean read1,read2).

VDRC number	Fly symbol	Human symbol	RNA concentration (ng/μl)	Volume (μl)	Total Mass (μg)	Clean reads	Clean bases	Q20(%)
6465	*ClC-c*	*CLCN3*	1.39	40	0.06	51,853,150	5,185,315,000	95.68%
12242	*Ctl1*	*SLC44A1*	10.3	40	0.41	52,239,002	5,223,900,200	95.94%
16137	*Hip1*	*HIP1R*	24.1	40	0.96	51,662,710	5,166,271,000	95.52%
22924	*Vha100-1*	*ATP6V0A1*	3.69	40	0.15	47,561,146	4,756,114,600	95.83%
24617	*EndoA*	*SH3GL2*	26.9	40	1.08	51,933,872	5,193,387,200	95.44%
25406	*Mccc1*	*MCCC1*	4.31	40	0.17	49,913,570	4,991,357,000	95.58%
26035	*CG7156*	*RPS6KL1*	10.3	40	0.41	50,451,936	5,045,193,600	95.58%
41259	*MICU3*	*MICU3*	11.6	40	0.46	54,369,786	5,436,978,600	95.72%
44825	*CG32066*	FAM49B	31.6	40	1.26	52,050,708	5,205,070,800	95.95%
45345	*CtsB1*	*CTSB*	7.87	40	0.31	52,103,894	5,210,389,400	95.78%
47868	*CG33181*	*SLC41A1*	16.3	40	0.65	51,782,270	5,178,227,000	95.38%
60000	Control line	NA	22	40	0.88	51,963,058	5,196,305,800	95.74%

Supplementary Table S4 | Within-gene read counts with between sample normalization using trimmed mean of M-values (TMM) of the 11 selected putative risk genes for sporadic PD. The column "$TMM_{UAS-GAL4}$" shows the TMM of the respective gene knockdown of the UAS-GAL4 lines, whereas the column "$TMM_{W1118-GAL4}$" shows the corresponding gene's TMM for the common control line. The column "Ratio" is the ratio between $TMM_{UAS-GAL4}$ and $TMM_{W1118-GAL4}$.

Fly gene symbol	$TMM_{UAS-GAL4}$	$TMM_{W1118-GAL4}$	Ratio	log2(Ratio)
Ctsb1	23.0225	84.3228	0.27	-1.87
CG7156	12.608	26.2477	0.48	-1.058
Mccc1	25.4293	43.815	0.58	-0.78
Hip1	31.8947	45.8818	0.70	-0.52
CG33181	126.467	176.293	0.72	-0.48
Vha100.1	129.217	177.945	0.73	-0.46
CG32066	50.3101	66.5491	0.76	-0.40
Ctl1	61.1542	69.6493	0.88	-0.19
MICU3	59.8264	65.331	0.92	-0.13
ClCc	140.981	131.018	1.08	0.11
EndoA	388.711	358.581	1.08	0.12

Supplementary Table S5 | Statistics from analysis of variance (ANOVA) within each *Drosophila* line investigating whether an effect of ageing on flight response was observed. The table contains for each test day the mean activity (Mean), standard deviation of the mean (SD), the number of observations (n, mean of five pictures for five flies within test vial), the regression coefficient (Beta), and the associated *P*-value from ANOVA.

	**Day 5**			**Day 10**			**Day 15**				
**Fly line**	**Mean**	**SD**	**n**	**Mean**	**SD**	**n**	**Mean**	**SD**	**n**	**Beta**	***P*-value**
Ctl1	2.34	1.26	45	1.76	1.03	45	1.92	1.34	45	-0.04	0.07
Hip1	2.86	1.14	59	2.63	1.13	60	3.1	1.08	60	0.03	0.18
Vha100-1	2.4	1.24	55	2.06	1.16	50	1.62	1.33	20	-0.07	0.03
Mccc1	2.84	0.97	69	2.82	1.19	73	2.75	1.14	70	-0.01	0.65
CG7156	2.67	1.24	69	2.49	1.19	70	2.04	0.96	65	-0.06	0.0018
MICU3	1.84	1.02	74	1.78	1.08	67	1.71	1.1	65	-0.01	0.4
CG32066	2.63	0.88	75	2.25	1.19	80	2.44	1.03	75	-0.02	0.28
CtsB1	2.84	1.12	84	2.17	1.24	80	1.17	1.1	45	-0.16	1.97×10^{-13}
CG33181	2.76	1.1	74	2.34	1.06	75	2.03	0.98	75	-0.07	4.6×10^{-6}
Control	2.73	1.03	84	2.82	1.21	90	2.63	1.13	85	-0.01	0.53

Supplementary Table S6 | Statistics from analysis of variance (ANOVA) comparing each *Drosophila* knockdown line with the common control line (w_{1118}-GAL4) at test day 5. The table contains the mean activity (Mean), standard deviation of the mean (SD), the number of observations (n, mean of five pictures for five flies within test vial), the difference between knockdown line and control, the standard error of the difference in activity (computed as $\sqrt{\frac{SD_{UAS-GAL4}}{n_{UAS-GAL4}} + \frac{SD_{W1118-GAL4}}{n_{W1118-GAL4}}}$), the %-reduction in activity compared to the control line, the associated *P*-value from ANOVA, and the false-discovery rate adjusted (FDR) *P*-value.

Test day	Fly line	Human gene	Mean	SD	n	Difference to w_{1118}	SE on difference	%-reduction to w_{1118}	*P*-value	FDR
5	*MICU3*	*MICU3*	1.84	1.02	74	-0.89	0.16	32.75	2.94E-07	2.65E-06
5	*Ctl1*	*SLC44A1*	2.34	1.26	45	-0.39	0.20	14.18	9.11E-02	4.10E-01
5	*Vha100-1*	*ATP6V0A1*	2.40	1.24	55	-0.33	0.19	11.95	1.85E-01	5.54E-01
5	*Hip1*	*HIP1R*	2.86	1.14	59	0.13	0.18	-4.65	5.83E-01	7.50E-01
5	*CtsB1*	*CTSB*	2.84	1.12	84	0.11	0.16	-4.17	4.04E-01	7.50E-01
5	*Mccc1*	*MCCC1*	2.84	0.97	69	0.10	0.16	-3.83	5.38E-01	7.50E-01
5	*CG32066*	*FAM49B*	2.63	0.88	75	-0.10	0.15	3.82	4.17E-01	7.50E-01
5	*CG7156*	*RPS6KL1*	2.67	1.24	69	-0.06	0.17	2.21	9.00E-01	9.00E-01
5	*CG33181*	*SLC41A1*	2.76	1.10	74	0.03	0.16	-0.97	8.14E-01	9.00E-01

Supplementary Table S7 | Statistics from analysis of variance (ANOVA) comparing each *Drosophila* knockdown line with the common control line (w_{1118}-GAL4) at test day 10. The table contains the mean activity (Mean), standard deviation of the mean (SD), the number of observations (n, mean of five pictures for five flies within test vial), the difference between knockdown line and control, the standard error of the difference in activity (computed as $\sqrt{\frac{SD_{UAS-GAL4}}{n_{UAS-GAL4}} + \frac{SD_{W1118-GAL4}}{n_{W1118-GAL4}}}$), the %-reduction in activity compared to the control line, the associated *P*-value from ANOVA, and the false-discovery rate (FDR) adjusted *P*-value.

Test day	Fly line	Human gene	Mean	SD	n	Difference to w_{1118}	SE on difference	%-reduction to w_{1118}	*P*-value	FDR
10	*Ctl1*	*SLC44A1*	1.757	1.031	45	-1.06	0.19	37.61	3.76E-07	1.69E-06
10	*MICU3*	*MICU3*	1.776	1.076	67	-1.04	0.17	36.93	1.04E-07	9.38E-07
10	*Vha100-1*	*ATP6V0A1*	2.056	1.159	50	-0.76	0.19	26.97	1.96E-03	4.42E-03
10	*CtsB1*	*CTSB*	2.171	1.244	80	-0.65	0.17	22.91	6.20E-05	1.86E-04
10	*CG32066*	*FAM49B*	2.251	1.186	80	-0.57	0.17	20.07	4.53E-03	8.16E-03
10	*CG33181*	*SLC41A1*	2.340	1.060	75	-0.48	0.17	16.92	2.17E-02	3.26E-02
10	*CG7156*	*RPS6KL1*	2.485	1.190	70	-0.33	0.17	11.74	4.33E-01	4.87E-01
10	*Hip1*	*HIP1R*	2.634	1.127	60	-0.18	0.18	6.47	4.06E-01	4.87E-01
10	*Mccc1*	*MCCC1*	2.825	1.187	73	0.01	0.17	-0.32	7.21E-01	7.21E-01

Supplementary Table S8 | Statistics from analysis of variance (ANOVA) comparing each *Drosophila* knockdown line with the common control line (w_{1118}-GAL4) at test day 15. The table contains the mean activity (Mean), standard deviation of the mean (SD), the number of observations (n, mean of five pictures for five flies within test vial), the difference between knockdown line and control, the standard error of the difference in activity (computed as $\sqrt{\frac{SD_{UAS-GAL4}}{n_{UAS-GAL4}} + \frac{SD_{W1118-GAL4}}{n_{W1118-GAL4}}}$), the %-reduction in activity compared to the control line, the associated *P*-value from ANOVA, and the false-discovery rate (FDR) adjusted *P*-value.

Test day	Fly line	Human gene	Mean	SD	n	Difference to w_{1118}	SE on difference	%-reduction to w_{1118}	*P*-value	FDR
15	*CtsB1*	*CTSB*	1.17	1.10	45	-1.46	0.19	55.59	1.11E-12	9.96E-12
15	*Vha100-1*	*ATP6V0A1*	1.62	1.33	20	-1.01	0.28	38.46	1.15E-01	1.47E-01
15	*MICU3*	*MICU3*	1.71	1.10	65	-0.92	0.17	34.90	1.31E-06	5.91E-06
15	*Ctl1*	*SLC44A1*	1.92	1.34	45	-0.70	0.21	26.77	9.11E-03	1.37E-02
15	*CG33181*	*SLC41A1*	2.03	0.98	75	-0.60	0.16	22.82	7.91E-04	1.78E-03
15	*CG7156*	*RPS6KL1*	2.04	0.96	65	-0.58	0.17	22.17	5.65E-03	1.02E-02
15	*Hip1*	*HIP1R*	3.10	1.08	60	0.48	0.18	-18.11	5.72E-04	1.72E-03
15	*CG32066*	*FAM49B*	2.44	1.03	75	-0.18	0.16	6.89	5.42E-01	5.42E-01
15	*Mccc1*	*MCCC1*	2.75	1.14	70	0.13	0.17	-4.87	1.68E-01	1.89E-01

**Supplementary Table S9 | ** Average survival from day 5 to day 10, and average survival from day 5 to day 15. The %-survival was computed as proportion of flies that survived from first test day (i.e., day 5) to test day 2 and test day 3 and is reported as the mean of the mean within replicate (*i.e.*, across technical replicates).

		Mean %-survival from	
**Line**	**Gene**	**Day 5 - 10**	**Day 5 - 15**
41259	*MICU3*	100	100
60000	*control*	100	100
44825	*CG32066*	100	100
6465	*ClC-c*	25	23
16137	*Hip1*	100	100
22924	*Vha100-1*	62	52
12242	*Ctl1*	100	99
45345	*CtsB1*	92	72
47868	*CG33181*	100	100
25406	*Mccc1*	100	99
26035	*CG7156*	99	99
